# Deep Learning with Dynamically Weighted Loss Function for Sensor-Based Prognostics and Health Management

**DOI:** 10.3390/s20030723

**Published:** 2020-01-28

**Authors:** Divish Rengasamy, Mina Jafari, Benjamin Rothwell, Xin Chen, Grazziela P. Figueredo

**Affiliations:** 1 Gas Turbine and Transmissions Research Centre, The University of Nottingham, Nottingham NG7 2RD, UK; benjamin.rothwell@nottingham.ac.uk; 2 Intelligent Modelling and Analysis Group, School of Computer Science, The University of Nottingham, Nottingham NG8 1BB, UK; mina.jafari@nottingham.ac.uk (M.J.); xin.chen@nottingham.ac.uk (X.C.); 3 The Advanced Data Analysis Centre, The University of Nottingham, Nottingham NG8 1BB, UK; grazziela.figueredo@nottingham.ac.uk

**Keywords:** predictive maintenance, deep learning, prognostics and health management, loss function, weighted loss function

## Abstract

Deep learning has been employed to prognostic and health management of automotive and aerospace with promising results. Literature in this area has revealed that most contributions regarding deep learning is largely focused on the model’s architecture. However, contributions regarding improvement of different aspects in deep learning, such as custom loss function for prognostic and health management are scarce. There is therefore an opportunity to improve upon the effectiveness of deep learning for the system’s prognostics and diagnostics without modifying the models’ architecture. To address this gap, the use of two different dynamically weighted loss functions, a newly proposed weighting mechanism and a focal loss function for prognostics and diagnostics task are investigated. A dynamically weighted loss function is expected to modify the learning process by augmenting the loss function with a weight value corresponding to the learning error of each data instance. The objective is to force deep learning models to focus on those instances where larger learning errors occur in order to improve their performance. The two loss functions used are evaluated using four popular deep learning architectures, namely, deep feedforward neural network, one-dimensional convolutional neural network, bidirectional gated recurrent unit and bidirectional long short-term memory on the commercial modular aero-propulsion system simulation data from NASA and air pressure system failure data for Scania trucks. Experimental results show that dynamically-weighted loss functions helps us achieve significant improvement for remaining useful life prediction and fault detection rate over non-weighted loss function predictions.

## 1. Introduction

Prognostic and health management (PHM) has become increasingly important in maintaining the integrity of automotive, aerospace, and manufacturing systems [1,2,3,4]. Machines are now fully equipped with sensors that constantly gather information regarding their status. The ability to utilize sensor data to accurately predict and diagnose problems facilitates effective maintenance management. In addition, the widespread use of sensors in machines has allowed for the transition from time-based maintenance to condition-based maintenance, where decision making is based on data collected via sensor monitoring, allowing for more flexible, reliable and robust maintenance services.

The increased number of successful examples of applications of deep learning in manufacturing, automotive, and aerospace industry has shown that it is a viable tool for condition-based maintenance [5,6,7,8,9]. Current deep learning research for these application areas, however, mostly focuses on changing the model architectures to improve remaining useful life (RUL) or fault prediction accuracy. The literature regarding improvement of other components of the deep learning model is scarce. Aspects such as custom loss function and hyperparameter optimization are not largely investigated in PHM research. In particular, this paper is interested in establishing the means to improve the standard loss function calculation to achieve better prediction accuracy. This approach modifies the loss function so that the weights associated with it are dynamically calculated. The error calculated from the dynamically weighted loss function can be viewed as a mechanism to force the learning process to focus on those instances that has the poorest prediction outcomes from the deep learning model. The objective is to improve the overall predictive performance of the deep learning systems investigated. The weighted loss function proposed works by generating a weight map [10], which is calculated based on the predicted value and error obtained for each instance. This method is therefore applicable to both prognostic and diagnostic tasks. In addition to the proposed weighted loss function, an existing dynamically weighted loss function, focal loss [11] (FL), that is designed for predicting probabilistic outputs which are more suited for diagnostics task such as fault detection were investigated. FL is an extension of cross entropy (CE) loss with dynamically weighted loss. The hypothesis is that the deep learning models using dynamically weighted loss function will learn more effectively compared to a standard loss function.

The performance of the new approach is examined by observing deep learning models’ predictive performance for two case studies: (1) Gas turbine engine remaining useful life (RUL) prediction using commercial modular aero-propulsion system simulation (CMAPSS) with the weighted loss function proposed in this paper and (2) air pressure system (APS) fault detection in trucks using the FL. CMAPSS is a run-to-failure gas turbine engine dataset openly sourced by NASA [12] and it is the standard dataset to compare different machine learning models for aerospace prognostics [13,14]. The APS fault detection dataset is collected from heavy Scania trucks in everyday usage. APS is a crucial part that helps in the braking and gear changing of trucks. The goal is to accurately detect the fault in APS and, most importantly, not to omit a fault when it is present. Results show that the proposed loss function and FL outperforms non-weighted loss function using deep feedforward neural network (DNN), one-dimensional convolutional neural network (CNN1D), bidirectional gated recurrent unit (Bi-GRU) and bidirectional long short-term memory (Bi-LSTM) deep learning architectures for the CMAPSS and APS results.

This paper is organized as follows. Section 2 provides a background on neural networks and their learning processes. In addition, it introduces the deep learning architectures used in this paper together with a review of their applications to PHM. Section 3 introduces our proposed weighted loss function as well as FL. Section 4 outlines the experimental design. Section 5 presents the results and discussions. Finally, the conclusions and future work are drawn in Section 6.

## 2. Background

This section provides a background on the basic structure of a neural network and how its learning process takes place. In addition, we introduce the deep learning architectures used in this paper and review how they are used for PHM. From our survey, we observed that most work focuses on modifying the networks’ architecture, with little focus given to the loss function.

### 2.1. How Neural Network Learning Is Performed

**Figure 1:**
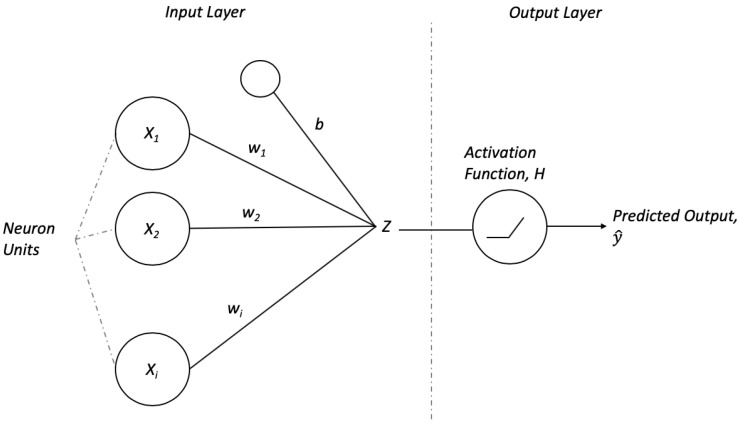
Basic components in a perceptron comprises of the input layer that can take in an arbitrary number of inputs, *s*, the weight, *w* that maps the inputs to the subsequent layer, a bias, *b*, activation function *H* to introduce non-linearity into the function and the output, *Z*.

To illustrate how the learning process of a neural network typically occurs, a simple architecture known as perceptron is employed. A perceptron consists of an input layer with its units as shown in Figure 1. Each neuron unit learns to assign a weight value (*w*) to each of its inputs (*x*). As expressed in Equation (1), the output is the sum of the multiplication of the inputs and their corresponding weights, and it is followed by adding a constant value bias, *b*. The inputs in the context of PHM are the sensors data collected and the output will produce a prediction according to the task at hand.

(1) $$Z_{\theta }\left(x_{i}\right)=x_{i}*w_{i}+b$$

where θ is the weight, *w* and bias, *b*. Subsequently, there is an activation function before the final output. The purpose of the activation function is to introduce non-linearity into the output of a neuron. To learn, first the weights $w_{i}$ and bias *b* are randomly initialized. A widely used activation function is the rectified linear unit (ReLU), as calculated in Equation (2).

(2) $$H_{\theta }\left(x_{i}\right)=max\left(0,Z\left(x_{i},\theta_{i}\right)\right)$$

Furthermore, the output from activation function is equivalent to the predicted output, $\hat{y_{i}}$,

(3) $$\hat{y_{i}}=H_{\theta }\left(x_{i}\right)$$

Once the predicted output, $\hat{y_{i}}$ is obtained, the error, *E* of prediction can be evaluated using the perceptron’s output against the actual value, $y_{i}$.

(4) $$E=\sum_{i=1}^{n}\left[\hat{y_{i}}-y_{i}\right]$$

The error, *E* calculated in (4) as an input to calculate the loss using a loss function. Equation (5) is a mean square loss function for this application.

(5) $$l\left(\theta_{i}\right)=\frac{1}{2n}\sum_{i=1}^{n}E^{2}$$

Subsequently, gradient descent is used to update the weights and biases based on the magnitude of the loss. Gradient descent is an iterative optimization algorithm used to minimize the loss by updating the weights as shown in Equation (6)

(6) $$w_{i}=w_{i}-\alpha \frac{\partial }{\partial w_{i}}l\left(w_{i}\right)$$

**Figure 2:**
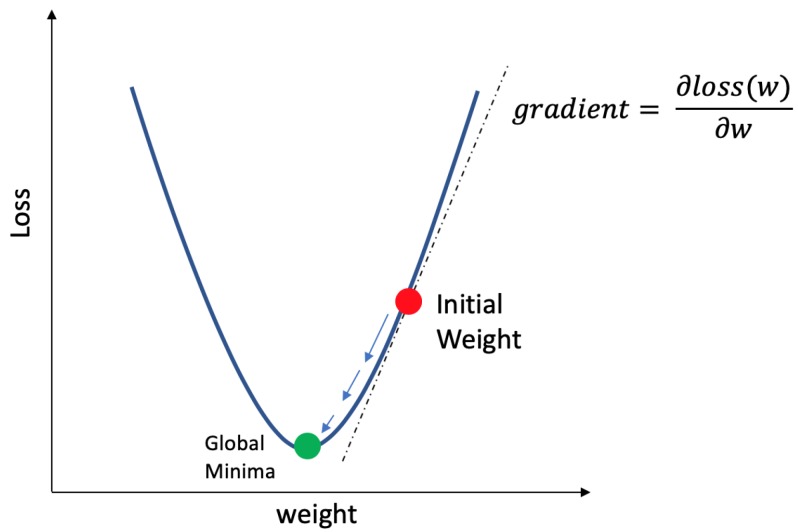
An initial weight value iteratively minimized based on the partial derivative of a loss function to achieve global minima in loss.

The partial derivative in Equation (6) takes the derivative of the loss function with respect to weight is the equivalent of calculating the gradient of loss. The learning rate α controls the magnitude of change in each iteration. Through the iteration, gradient descent will converge on the minima and provide the best value for each weight parameter as illustrated in Figure 2.

### 2.2. Deep Feedforward Neural Network

**Figure 3:**
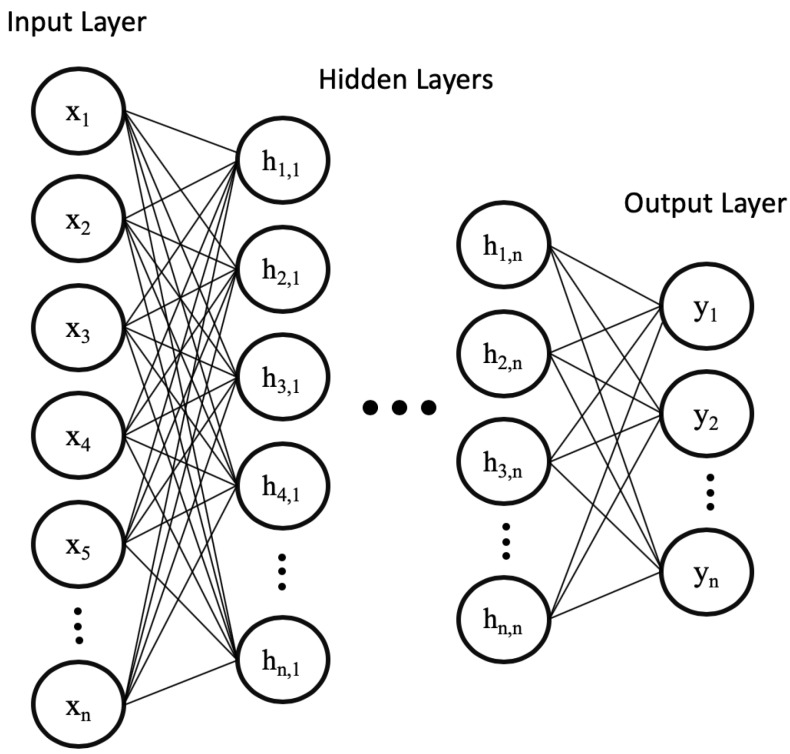
A deep feedforward neural network (DNN) similar to the perceptron has the input layer along with the output layer. However, the DNN has a large number of hidden layers and neuron units.

Early form of feedforward neural network are multi-layer perceptron (MLP). A MLP consists of three layer types, namely the input, hidden, and the output layer. Each layer is composed of several neuron units. The neurons in each layer are fully connected to the neurons in the subsequent layer and the connection holds a weight value that will contribute the output value. The connections’ weights are randomly initialized and then updated using the gradient descent method (introduced in Section 2.1) during training. As shown in Figure 3, the MLP neural network can be extended to a deep neural network by increasing the number of hidden layers, which allows for learning more complicated relationships between inputs.

### 2.3. Convolutional Neural Networks

**Figure 4:**
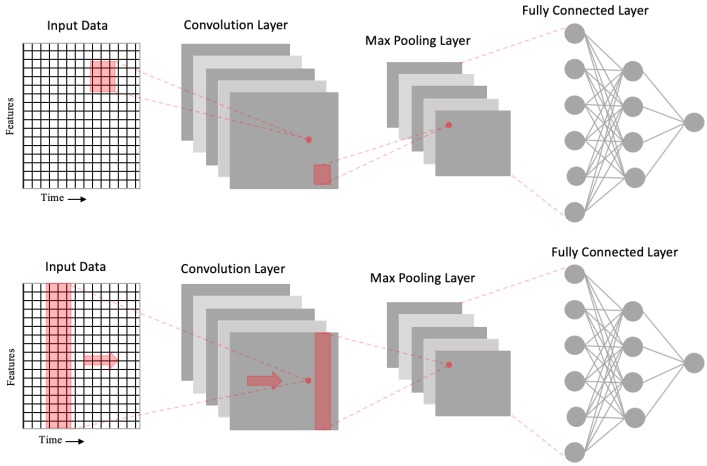
The **top** part of the figure illustrates a normal two dimensional convolutional neural network (CNN) with its convolutional layer and max pooling layer. The max pooling layer is subsequently flattened to feed the data into a fully connected layer. The **bottom** figure is a one-dimensional convolutional neural network (CNN1D) network where the filter is moving in only one direction to perform the convolution and max-pooling operations.

Convolutional neural networks (CNNs) [15] are neural networks that contain different layers such as the convolution, max pooling, and fully connected layer. The purpose of max pooling layer is to downsample the input and reduce the dimension while the convolution layer extracts high-level features from the input. This allows CNN to perform better on data that has high spatial correlation with its neighborhood data-points. Figure 4 shows how the spatial relationship within data are preserved through convolution and max pooling using a filter. Furthermore, CNN1D uses a filter that is the same height as the input and the convolution operation occur in a single direction as illustrated in the bottom part of Figure 4.

### 2.4. Long Short-Term Memory

**Figure 5:**
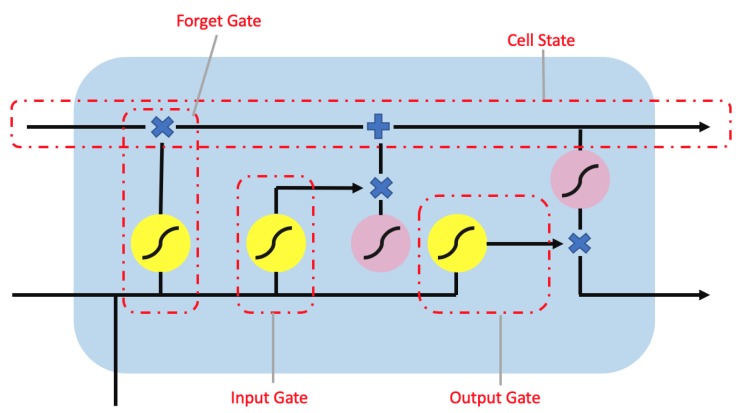
The long short-term memory (LSTM) unit contain a forget gate, output gate and input gate. The yellow circle represents the sigmoid activation function while the pink circle represents a tanh activation function. Additionally, the ”×” and “+” symbols are the element-wise multiplication and addition operator.

**Figure 6:**
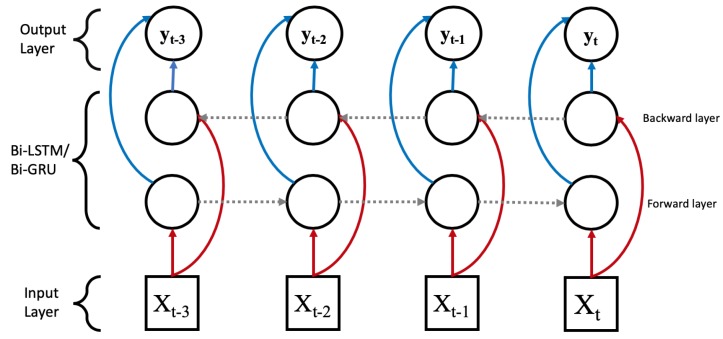
The Bi-LSTM and Bi-GRU are structurally the same except for the LSTM and GRU unit. The red arrows indicate the input value flow, blue arrows are the output values, and the grey arrows represent the information flow between the LSTM/GRU units.

The long short-term memory (LSTM) network [16] is a variant of the recurrent neural network [17] (RNN) designed with chain units consisting of input, forget, and output gates as shown in Figure 5. Gates are responsible for regulating what information is passed through to the next unit. The input gate controls the influence of the current input. The forget gate within each unit controls how much information needs to be retained. The output gate controls whether the flow is passed on to the next LSTM unit. This architecture allows for the learning of data with long-term dependencies. Furthermore, a bidirectional LSTM as shown in Figure 6 connects two hidden layer of LSTM in the opposite direction to increase the information available to the network by using the past and future states.

### 2.5. Gated Recurrent Unit

**Figure 7:**
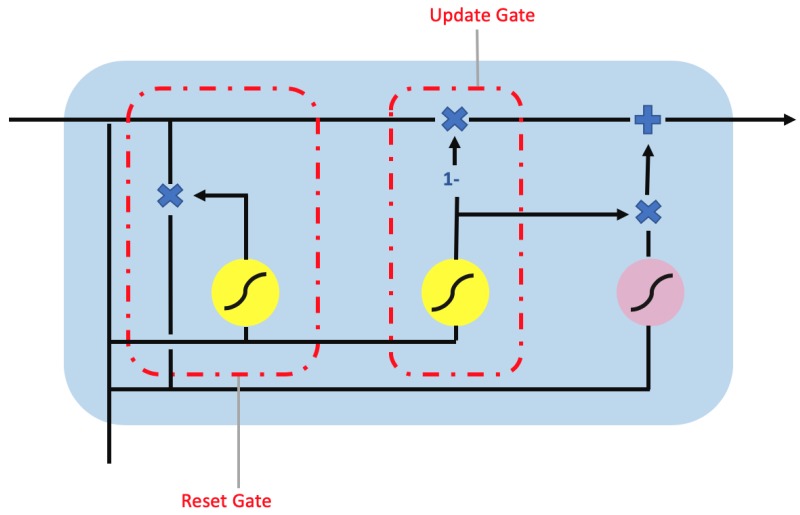
The gated recurrent unit (GRU) unit contain a reset gate and update gate. The yellow circle represents the sigmoid activation function while the pink circle represents a tanh activation function. Additionally, the ”×”, “+”, and ”1–” symbols are the element-wise multiplication, addition, and inversion operator.

The gated recurrent unit (GRU) [18] is proposed as alternative to LSTM. While GRU and LSTM are similar, they differs in the number of parameters and type of gates. GRU uses only two gates as shown in Figure 7. The two gates are (1) the reset gate to control the memory retention from previous unit and addition of new memory into the unit and (2) the update gate to control input and to remove new information. Therefore, GRU has fewer parameters in its design than LSTM, thus reducing the model complexity while improving on computational efficiency. Similar to LSTM, GRU can be extended to Bi-GRU as shown in Figure 6.

### 2.6. Current Deep Learning Solutions

Tamilselvan et al. [19] uses a deep belief network (DBN) to identify the health state of aero-engine also using CMAPSS. The DBN classifier used consists of three hidden-layers. The conjugate gradient approach from Hinton et al. [20] is used to fine-tune the DBN classifier after it has been pretrained and trained. The DBN fault classification of aero-engines is compared to SVM, backpropagation neural network (BNN), self-organizing maps [21] and Mahalanobis distance. Results show that DBN achieves the best classification accuracy for five of the six operating conditions.

To further extend the capability of DBN, Zhang et al. [22] apply multiobjective deep belief networks ensemble (MODBNE) to CMAPSS. The trained DBNs aimed at minimizing of the DBNs prediction error and maximizing the diversity of outputs between DBNs. The optimized DBNs are combined using single-objective differential evolution to create the ensemble. Results show that MODBNE achieves the most accurate estimation of RUL when compared to 10 other data-driven methods, e.g., DBN, sequential Kalman filter, MLP, extreme learning machine (ELM), hierarchical ELM, SVM, LASSO, extra tree regressor, k neighbours regressor, gradient boosting and random forest.

LSTM is a popular architecture choice for sensor data as it is specially designed to perform predictions on sequential data such as text and time series data. Yuan et al. [23] and Zheng et al. [24] employ LSTM to predict RUL on CMAPSS. Both groups of authors convert the RUL to piece-wise RUL. Initially, the RUL is set to a constant value to mimic the condition before degradation and subsequently it linearly decreases to show degradation. LSTM are compared with standard RNN, GRU AdaBoost LSTM, CNN, SVM, relevance vector regression (RVR), and MLP. The results reveal that the LSTM outperforms all other methods investigated for both RUL estimation and fault occurrence predictions. Ellefsen et al. [25] first uses the restricted Boltzmann machine (RBM) to pretrain the model in an unsupervised manner to automatically generate new degradation-related features from the raw data. Subsequently, the newly generated features are used as input for LSTM to predict the RUL. The hyperparameters of the model are tuned and optimized by Genetic Algorithms (GA). The results showed that the combination of RBM and LSTM achieves the state-of-the-art score function (SF) and root mean squared error (RMSE) (Wang et al. [26]). The authors showed that Bi-LSTM’s hidden layers are able to implicitly extract degradation features without unsupervised pretraining of the model. The results obtained from Bi-LSTM without pretraining and GA optimized hyperparameter were similar to the state-of-the-art performance.

Furthermore, Babu et al. [27], through using the CMAPSS dataset, showed that CNN increases prediction accuracy when compared results to MLP, SVM, and RVR. Li et al. [28] used deep CNN to estimate both the RUL and fault diagnosis of aircraft turbofan engines. The authors employed a conventional CNN and training was conducted using mini-batch gradient descent [29]. Results from the CNN were compared to LSTM, RULCLIPPER [14], random forest, gradient boosting, SVM, echo state network with Kalman filter [30], multi-objective deep belief networks ensemble [22] and time window-based NN [31]. Results revealed that CNN outperforms the LSTM, RNN, and DNN for RMSE. The authors also showed that training time increases proportionally to the number of convolutional layers, and concluded that the optimal number of layers for their problem is five.

## 3. Dynamically Weighted Loss Function

This section discusses how the proposed loss function and FL are constructed mathematically and the reasoning behind these methods.

### 3.1. Proposed Dynamically Weighted Loss Function

In machine learning, the loss function is the difference between the ground truth and the predicted output of the model. The goal of the learning algorithm is to minimize the error produced by the loss function during training. For the first case study of predicting RUL of gas turbine engine degradation, mean square error (MSE) was selected as the choice of loss function as it is more suitable for the regression task. Other types of loss function for regression task include mean absolute error (MAE) and Huber loss. However, the gradient of MAE loss remained constant throughout training and did not decrease when loss was close to zero, making it unsuitable for a neural network to learn, as the large gradient could miss the minima as the error approaches zero. Furthermore, Huber loss was not chosen as the loss function as it required tuning of the hyperparameter. This introduces additional complexity when losses are dynamically weighted. The MSE loss function can be represented mathematically as,

(7) $$l\left(f\left(x\right),y\right)={\left(f\left(x\right)-y\right)}^{2}$$

where f(x) is the model output and *x* is input while *y* is the ground truth label. Next, the MSE is simply multiplied by a weight variable *D* to be converted to a weighted MSE.

(8) $$l\left(f\left(x\right),y\right)=D*{\left(f\left(x\right)-y\right)}^{2}$$

As mentioned in Section 2.1, a large error is an indication of poor learning on a particular instance in the dataset. To place more importance on instances with larger error, the weight variable from Equation (8) is updated to a function of f(x) and *y* as follows,

(9) $$l\left(f\left(x\right),y\right)=D\left(f\left(x\right),y\right)*{\left(f\left(x\right)-y\right)}^{2}$$

The specific weight used in this paper scale according to the following condition,

(10) $$D\left(f\left(x\right),y\right)=\left\{\begin{array}{cc} \frac{\left|f(x)-y\right|}{2} & \;\mathrm{if}\;|f(x)-y|\;\mathrm{is}\;<\;C\\ \left|f(x)-y\right| & \;\mathrm{otherwise}\; \end{array}\right.$$

The weighting is halved when the absolute difference between predicted value and ground truth is less than a particular constant, *C*. The constant is set to 10 as it is assumed that the model has learned this particular instances. In addition, the weight function can be generalized to take in different input on different loss function.

(11) $$l{\left(\theta ,f\left(x\right),y\right)}^{\prime }=D\left(\theta \right)*l\left(f\left(x\right),y\right)$$

**Figure 8:**
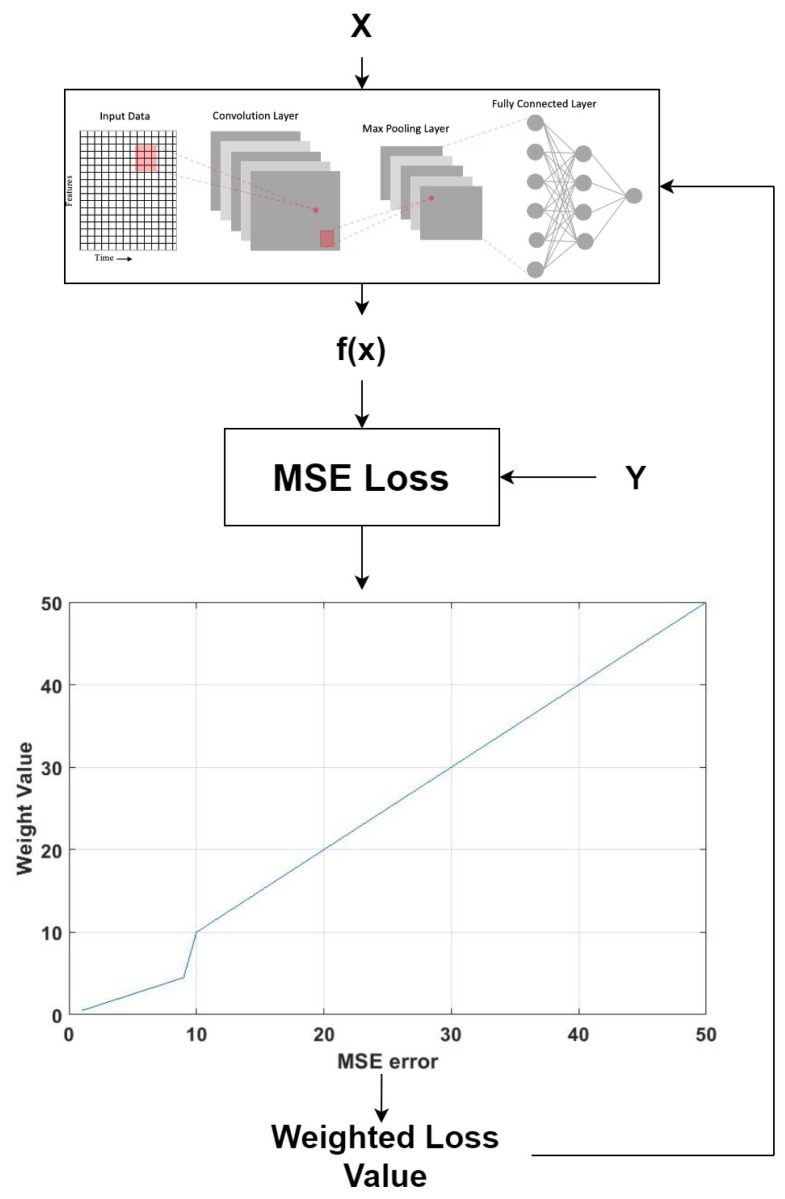
The output f(x) from the deep learning model and the ground truth *Y* are used to calculate the mean square error (MSE) for one instance. The MSE is then passed through a non-linear function to produce the weight that will be used to dynamically adjust the loss function.

The overall flow of the data from input to the new loss function is shown in Figure 8.

### 3.2. Focal Loss Function

For many fault detection tasks, the goal is to predict the fault and non-faulty condition given the sensors value. In essence, this is a binary classification problem. A deep learning model typically produces a probability value for each class using the softmax activation function, and the loss is calculated using the CE loss function. CE loss is a measure of the difference between two probability distributions. The first probability distribution is the actual class where the known class label has a probability of 1.0 and there is a probability of 0.0 for all other class labels. Subsequently, the second probability distribution is the predicted probability for each class. The CE loss function for binary classification can be represented mathematically as follows:

(12) CE(y,p)=−(y∗log(p)+(1−y)∗log(1−p))

where *p* is the deep learning model probabilistic output that ranges from [0,1] and *y* is the ground truth class either 0 or 1. Equation (12) can be simplified to the form:

(13) $$CE\left(y,p\right)=\left\{\begin{array}{cc} -log(p) & \;\mathrm{if}\;y=1\\ -log(1-p) & \;\mathrm{otherwise}.\; \end{array}\right.$$

To further simplify the CE loss function, $p_{o}$ can be defined as:

(14) $$p_{o}=\left\{\begin{array}{cc} p & \;\mathrm{if}\;y=1\\ \left(1-p\right) & \;\mathrm{otherwise}.\; \end{array}\right.$$

**Figure 9:**
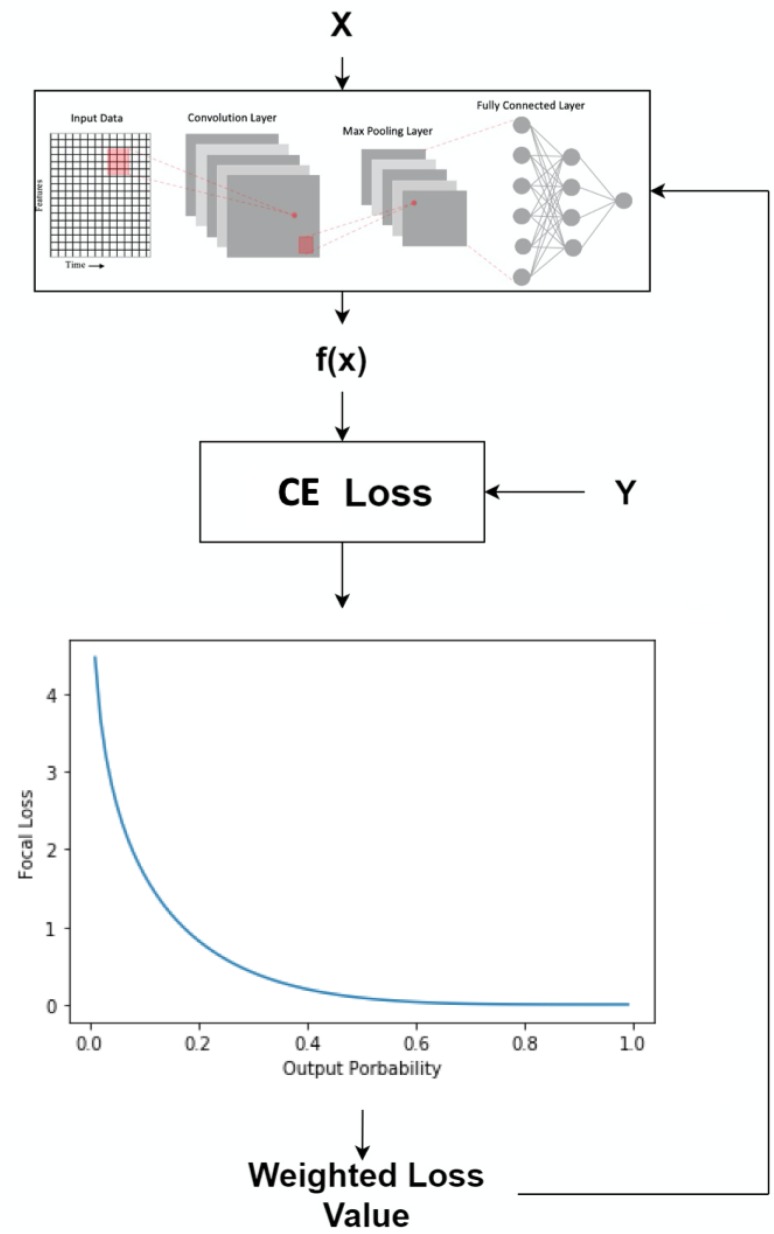
The output f(x) from the deep learning model and the ground truth *Y* are used to calculate the cross entropy (CE) loss for one instance. The CE is then combined with the weighted function to produce the weight that will be used to dynamically adjust the loss function.

Therefore, $CE\left(p_{o}\right)=-log\left(p_{o}\right)$. Subsequently, a weighting factor term is added to convert the CE loss function to FL,

(15) $$FL\left(p_{o}\right)=-\alpha_{o}{\left(1-p_{0}\right)}^{\gamma }log\left(p_{o}\right)$$

where α is a value between [0,1] and γ≥0. Both α and γ are tunable hyperparameters to optimize the performance of deep learning models. The α is a weighting parameter used to control the class imbalance problem. Additionally, the γ is a focusing parameter that controls the loss. Larger values of γ correspond to larger losses for badly learned instances. The data flow for the FL is the same as the proposed loss function in Section 3.1 and is it summarized in Figure 9.

## 4. Experimental Design

This section introduces the experimental design for both case studies, i.e., CMAPSS and APS truck fault data. The data preprocessing, deep learning architectures employed and the evaluation metrics step are described in this section.

### 4.1. Case Study 1: Remaining Useful Life Prediction of Gas Turbine Engine

#### 4.1.1. Data Description

The gas turbine engine degradation data used in this paper is CMAPSS by Saxena and Goebel [12]. The data were established from a high fidelity simulation of a complex non-linear system that closely models a real aerospace engine. The dataset contains one training set and one test set with an operating condition and fault pattern. The training set is the complete engine life cycle data, i.e., run To failure, but the testing set does not reach failure. The dataset consists of the engine unit number, the operating cycle number of each unit, the operating settings and the raw sensor measurements. The raw sensor features are shown in Table 1.

#### 4.1.2. Data Preprocessing

The CMAPSS data consists of 3 operation settings and 21 sensors features. However, a total of eight features are discarded as they remained constant throughout the gas turbine engine degradation process and provided no useful information. Additionally, the data is normalized between [0,1] to ensure that each feature is represented equally in the learning process. Subsequently, the labels are preprocessed. The labels are the remaining RUL cycle for each instance of the data and each complete cycle is degraded linearly. Since the fault does not occur at the early stages of engine cycle, the value of the maximum cycle is capped at 120 and remains constant until degradation has occurred. This allows the models to differentiate between the healthy state, a RUL of 120 and under degradation, and a RUL cycle of less than 120.

#### 4.1.3. Deep Learning Architectures Investigated

The following deep learning model architectures are employed to test the loss function in Equation (11), (1) bidirectional LSTM, (2) DNN, (3) CNN1D, and (4) bidirectional GRU. Their hyperparameters are listed in Table 2.

An L2 regularizer is added to the layers of all model shown in Table 2 to reduce overfitting. In the context of neural network, an L2 regularizer is mathematically equivalent to weight decays. The L2 regularizer prevents overfitting by limiting the complexity of the network through the penalization of larger weights, which keeps the weights smaller. Additionally, a dropout [32] rate of 0.5 is also added to all models tested to mitigate overfitting. Dropout is a technique for regularizing the network by randomly setting the output to zero (equivalent to setting the weight of the unit to 0).

#### 4.1.4. Evaluation Metrics

Performance evaluation is the key step to identify and compare the performance between different methods. NASA published a preferred method of performance evaluation for CMAPSS using the idea of asymmetric scoring. In the context of predictive maintenance, it is desirable to predict the time of failure early. Therefore, the scoring is asymmetric around the true time of failure such that late predictions are more heavily penalized than early predictions. The asymmetric scoring function is as follows:

(16) $$\mathrm{Scoring}\;\mathrm{Function}=\left\{\begin{array}{cc} \sum_{i=1}^{n}e^{\frac{-d}{10}}-1 & \;\mathrm{if}\;d<0\\ \sum_{i=1}^{n}e^{\frac{d}{13}}-1 & \;\mathrm{if}\;d\geq 0 \end{array}\right.$$

where *d* is f(x)−y. In addition, RMSE was employed as the second evaluation metrics:

(17) $$RMSE=\sqrt{\frac{1}{N}\sum_{i=1}^{N}{\left(f\left(x_{i}\right)-y_{i}\right)}^{2}}$$

The scoring function (SF) along with RMSE provides a suitable measure of the different deep learning models’ accuracy.

The evaluation metrics alone do not indicate if a result improvement is statistically significant or not. Therefore, the Mann–Whitney–Wilcoxon non-parametric test is used at a 0.05 significance level to test if result improvements are significant. The Mann–Whitney–Wilcoxon non-parametric test is employed for this test because the two results distribution are independent. In addition, the experiment is run 20 times to ensure that the results are more reliable and accurately represent the true distribution.

### 4.2. Case Study 2: Fault Detection in Air Pressure System of Heavy Trucks

#### 4.2.1. Data Description

The function of an APS is to produce pressurized air for braking and gear changes. Therefore, it is important that the APS’ fault are accurately detected as a miss in a truck’s fault could lead to undesirable outcome. The APS failure data has a total of 171 features from sensors on the truck. However, the name for the features have been anonymized for proprietary reasons. The training data consists of 60,000 instances of which 59,000 belong to the negative class (no fault) and 1000 to the positive class (fault present). As for the testing data, it consists of 16,000 instances of which 15,625 belong to the no fault class and 375 to the fault class. The number of instances between the positive class and negative class is highly imbalanced as shown in Table 3.

#### 4.2.2. Data Preprocessing

The APS dataset contains 170 features and a binary class (True or False) as labels. The missing data are imputed using k-nearest neighbour (KNN) [33]. Furthermore, the synthetic minority over-sampling technique (SMOTE) is used to re-balance the training set as it is highly imbalanced as shown in Table 3. SMOTE is a way of increasing the minority class without directly duplicating instances of the minority class. Instead, new instances are synthesized within the clusters of minority classes. The reason this dataset is balanced for this experiment is because the goal is to investigate the effect of a dynamically weighted loss function on instances that are difficult to learn. Therefore, the balanced data is tested on deep learning models using normal and dynamically weighted loss functions. Further study is required to study the effect of dynamically weighted loss function on a highly imbalanced dataset.

#### 4.2.3. Deep Learning Architectures

**Figure 10:**
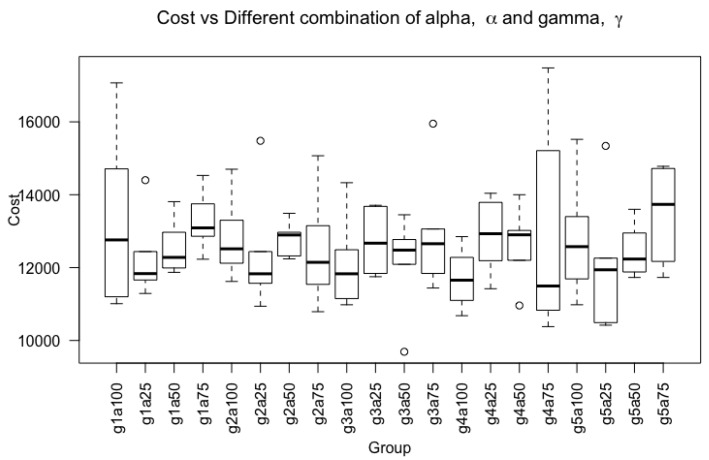
Boxplot of final cost using a combination of gamma value, [1, 2, 3, 4, 5] and alpha value, [0.25, 0.5, 0.75, 1.0]. The x-axis are denoted by the combination of alpha and gamma. For instance, ’g1a100’ represents gamma value of 1 and alpha of 1.0.

For consistency, the same deep learning architectures listed in Section 4.1.3 are used to test the FL shown in Equation (15). Their respective hyperparameters are listed in Table 4. Furthermore, the strategy adopted for mitigation of overfitting is the same as the technique described in Section 4.1.3 using a combination of L2 regularizer and drop rate of 0.5. In addition, the γ and α set for focal loss are 5 and 0.75 respectively. The γ and α value were experimented using a combination of γ=[1,2,3,4,5] and α=[0.25,0.5,0.75,1.0]. The results are shown in Figure 10 using a boxplot with different values of alpha and gamma. Additionally, it was ran six times to obtain the distribution. By using analysis of variance (ANOVA), it was found that the cost calculated using Equation (18) using different combinations of γ and α were not statistically significant different as it has the F-value of F(19,100)=0.588 and a *p*-value greater than 0.05 at p=0.907.

#### 4.2.4. Evaluation Metrics

**Figure 11:**
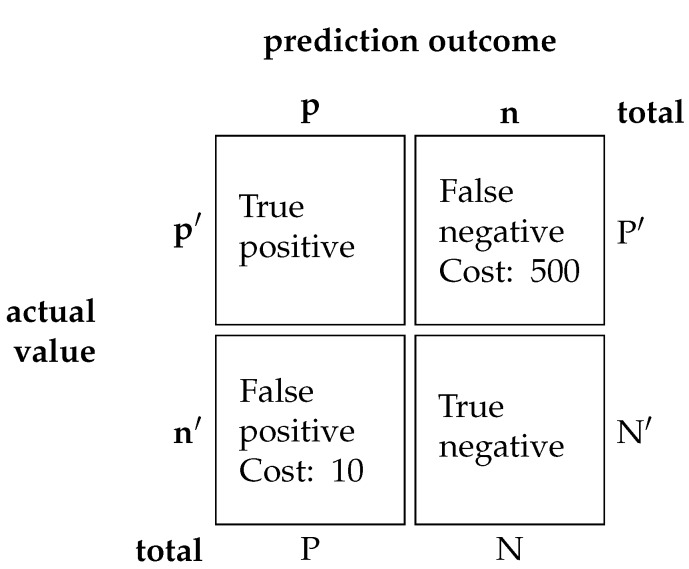
A confusion matrix with the associated cost of each fault. A confusion matrix tabulates the performance of a classification model. A true positive and true negative are correct classification therefore, there are no cost associated to it. Whereas false positive and false negative receive a cost of 10 and 500 respectively. The *p* and *n* represents positive and negative class while *P* and *N* represents the total positive and negative class. The actual class is denoted by an apostrophe.

The authors of the APS dataset from Scania published a cost-metric of misclassification as an evaluation metric. Binary classification has two faults: (1) False positive and (2) false negative. Each misclassification has a cost associated with it. In the context of the PHM of trucks, a false negative outcome has a more severe consequence compared to false positive outcome, and leads to an asymmetry in cost. A cost value of 10 and 500 are assigned to the false positive and false negative outcomes respectively to signify the asymmetry in cost. The cost value for the false positive and negative are specified by the data owner. The origin for the specific value of 10 and 500 are not explained. The total cost can be summarized as follows in Equation (18) and Figure 11:

(18) TotalCost=(Cost1∗Numberoffalsepositive)+(Cost2∗Numberoffalsenegative)

The goal is to minimize the cost. A large percentage of the cost factor comes from the false negative classification. Additionally, metrics such as false negative rate (FNR), false omission rate (FOR), and recall are also used. The formula for FNR, FOR and Recall is as follows:

(19) $$\mathrm{False}\mathrm{Negative}\mathrm{Rate}=\frac{\mathrm{False}\;\mathrm{Negative}}{\mathrm{True}\;\mathrm{Positive}+\mathrm{False}\;\mathrm{Negative}}$$

(20) $$\mathrm{False}\mathrm{Omission}\mathrm{Rate}=\frac{\mathrm{False}\;\mathrm{Negative}}{\mathrm{True}\;\mathrm{Negative}+\mathrm{False}\;\mathrm{Negative}}$$

(21) $$\mathrm{Recall}=\frac{\mathrm{True}\;\mathrm{Positive}}{\mathrm{True}\;\mathrm{Positive}+\mathrm{False}\;\mathrm{Negative}}$$

Finally, a precision-recall (PR) curve is added to study the relationship between precision and recall. Precision is the measurement for the false positive rate while recall is the rate of true positive against false negative. A PR curve is employed here as opposed to a receiver operating characteristic curve as the latter can be easily misinterpreted in highly imbalanced dataset [34].

## 5. Results and Discussion

This section discusses the results obtained using the methodology in Section 3 and experimental setup in Section 4.

### 5.1. Case Study 1: Remaining Useful Life Prediction of Gas Turbine Engine

Table 5 shows the comparison between the RUL prediction results of deep learning models with and without dynamically weighted loss function. Dynamically weighted loss function improved the scoring function’s values for all models tested. However, using the RMSE metric, Bi-LSTM and CNN1D showed improved performance while DNN and Bi-GRU’s result worsened. The DNN and Bi-GRU models with dynamically weighted loss function predicted earlier RUL, which caused the predicted output to deviate further from the ground truth but still showed an improvement in scoring function. This is due to the scoring function’s asymmetric property that resulted in the score favoring an early RUL prediction. The results shown in Table 5 arethe median values, and they do not include outliers.

**Figure 12:**
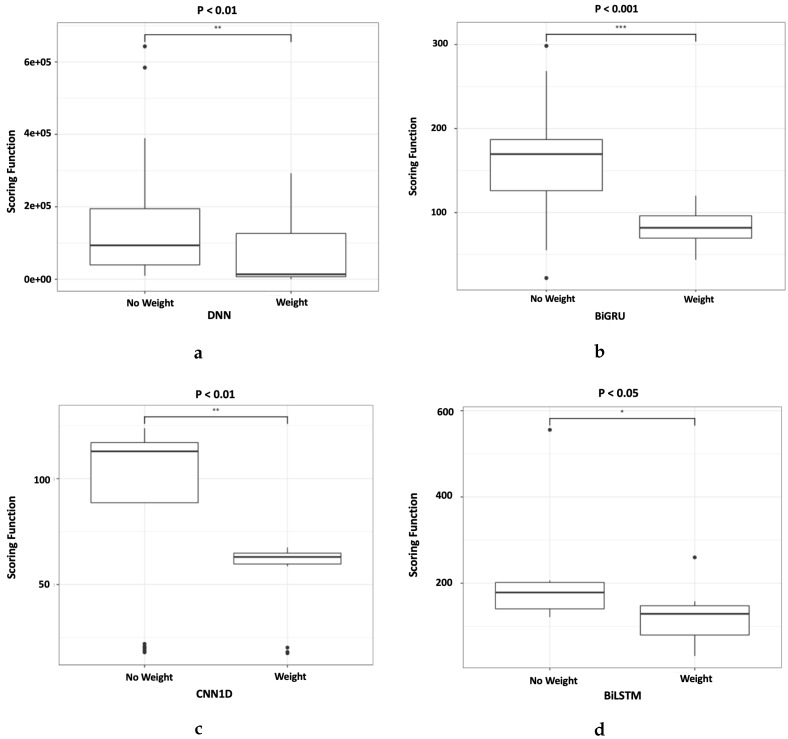
Boxplots of all scoring functions result from the four deep learning models, (**a**) DNN, (**b**) Bi-GRU, (**c**) CNN1D, and (d) Bi-LSTM using a dynamically weighted loss function, and without the weight. The asterisk on the top of each boxplot denotes the *p*-value where “***” < 0.001, “**” < 0.01, “*” < 0.05.

Subsequently, Figure 12 shows that the improvement made by using the new loss function is statistically significant. The number of “*” in Figure 12 represents the *p*-value where “***” < 0.001, “**” < 0.01, “*” < 0.05. Figure 12 clearly shows that the four models, DNN, Bi-GRU, CNN1D and Bi-LSTM results improvement are statistically significant. Bi-GRU has a *p*-value of <0.001, DNN and CNN1D has a *p*-value of <0.01, while Bi-LSTM has a *p*-value of <0.05. In addition, some anomalies are noted in the results as shown in the boxplots. This is caused by the random initialization of the initial weights which resulted in the variability of the final output. Therefore it is important to run the experiment multiple times to ensure that the true distribution of the final output is captured.

### 5.2. Case Study 2: Fault Detection in Air Pressure System of Heavy Trucks

Table 6 shows the cost, FNR, FOR, and recall for the deep learning architectures Bi-LSTM, DNN, CNN1D, and Bi-GRU using CE and FL. Bi-LSTM, DNN, CNN1D, and Bi-GRU with FL showed significant improvement across the cost, FNR, FOR, and recall metrics. CNN1D with FL achieved the lowest cost of 12,580 while Bi-GRU with cross entropy loss achieved the highest cost of 35,480. Furthermore, when FL was used as the choice of loss function the cost metric improved by an average of 31.5% across the tested models.

**Figure 13:**
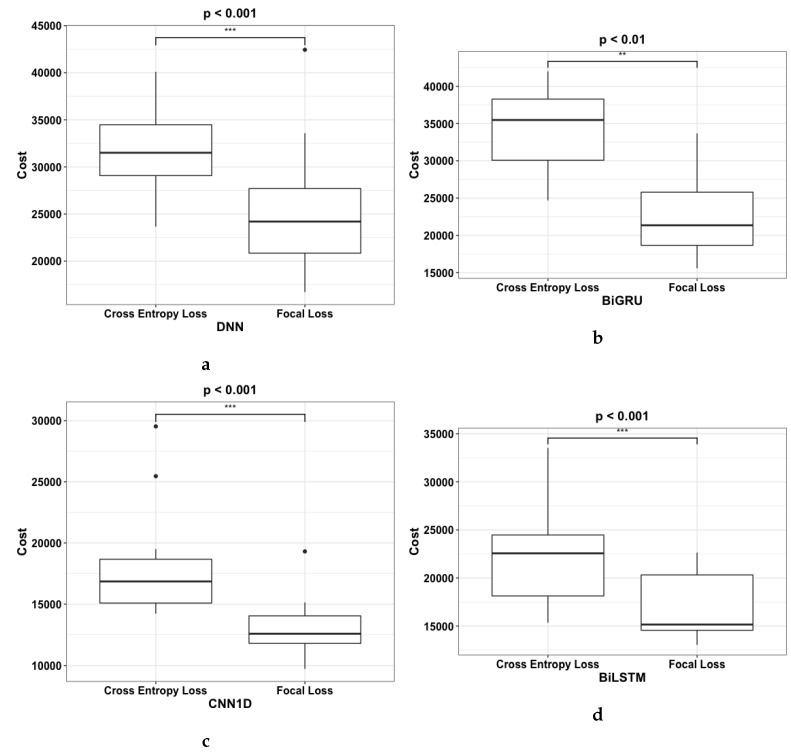
Boxplots of Costs result from using (**a**) DNN, (**b**) BiGRU, (**c**) CNN1D, and (d) BiLSTM with CE and FL respectively. The asterisk on the top of each boxplot denotes the *p*-value where “***” < 0.001, “**” < 0.01, “*” < 0.05.

The results of cost metric were plotted to show the distribution of output across 20 experimental runs as shown in Figure 13d. Similar to Section 5.1, the number of “*” in Figure 13d represents the *p*-value where “***” < 0.001, “**” < 0.01, “*” < 0.05. The boxplots show that using deep learning models with FL as the loss function resulted in improvements that were statistically significant. DNN, CNN1D, and Bi-LSTM had *p*-values of <0.001 while Bi-GRU had a *p*-value of <0.01. In addition, the anomalies within the experimental runs for each deep learning models are shown in the boxplots as black dot.

**Figure 14:**
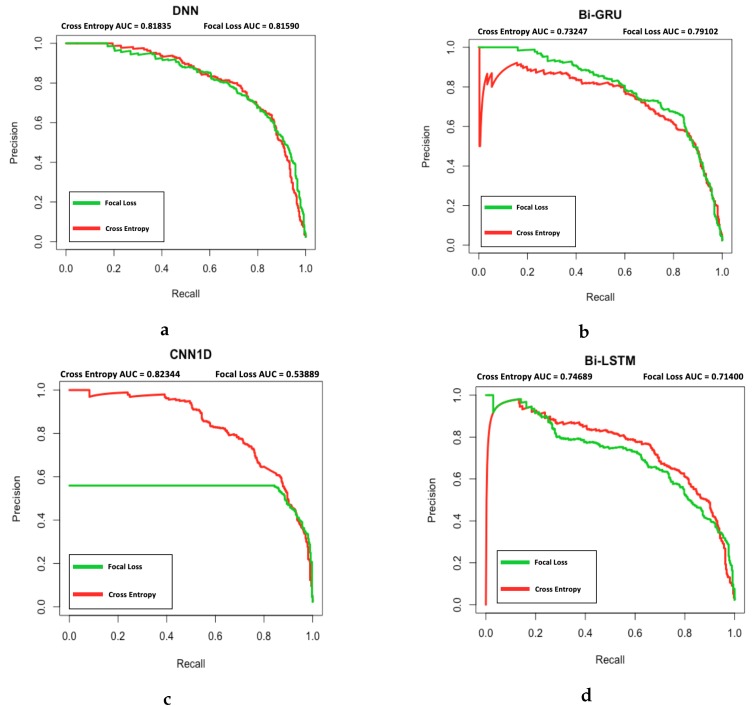
PR Curve for (**a**) DNN, (**b**) Bi-GRU, (**c**) CNN1D, and (**d**) Bi-LSTM using focal loss (Green line) vs. cross entropy loss (Red line). The AUC of PR curves are included at the top of each plot for each loss function.

Figure 14 displays the PR Curve for both FL and CE used with DNN, Bi-GRU, CNN1D, and Bi-LSTM. From the plot it can be observed that DNN’s PR curve and area under curve (AUC) for FL and CE are similar. Subsequently, Bi-GRU with FL has a higher AUC compared to Bi-GRU with CE and overall achieved higher precision for a given recall. Next, CNN1D and Bi-LSTM both had lower AUC when FL was used. CNN1D and Bi-LSTM both had a lower precision to achieve the same recall, with CNN1D being more extreme. This was caused by the overwhelming false positive prediction to achieve the low false negative count. However, as mentioned in Section 4.2.4 the cost of a false positive is significantly lower than a false negative, at a ratio of 10:500. Therefore, when the actual cost is accounted for as shown in Table 6 deep learning models with FL still outperformed CE in all cases.

## 6. Conclusions and Future Work

This paper demonstrated that the PHM of a gas turbine engine and APS system were improved by using deep learning models with a dynamically weighted loss function that focused on instances that were poorly learned during the training process. The proposed loss function and FL are aimed at increasing prognostics and diagnostics accuracy by improving on the existing loss function while keeping the deep learning architecture unchanged. We were able to show improvement in the RUL prediction accuracy on the CMAPSS dataset and fault detection on the APS truck failure dataset classification performance using four different deep learning architectures, e.g., DNN, CNN1D, Bi-GRU, and Bi-LSTM, when the dynamically weighted loss function was used. Subsequently, the results were validated by performing a Mann–Whitney–Wilcoxon non-parametric statistical significance test, which showed the main evaluation metric, being function for case study 1 and cost for case study 2. All deep learning architectures tested in this paper achieved statistically significant improvement (*p* < 0.05) when the dynamically weighted loss function was employed.

For future work, we consider improving the weighting function to perform better on PHM tasks. Furthermore, more analysis will be conducted to study the effect of the dynamically weighted loss function on an imbalance PHM dataset. In addition, we will investigate the usage of physics-based loss functions to create deep learning models with output that are scientifically consistent with the data. Finally, we will test the improved loss function with a different PHM dataset in aerospace and automotive applications.

## Figures and Tables

**Table 1:** Description of the commercial modular aero-propulsion system simulation (CMAPSS) dataset sensor features.

Symbol	Description	Unit
T2	Total temperature at fan inlet	°R
T24	Total temperature at Low Pressure Compressor outlet	°R
T30	Total temperature at High Pressure Compressor (HPC) outlet	°R
T50	Total temperature at Low Pressure Turbine outlet	°R
P2	Pressure at fan inlet	psia
P15	Total pressure in bypass-duct	psia
P30	Total pressure at HPC outlet	psia
Nf	Physical fan speed	rpm
Nc	Physical core speed	rpm
epr	Engine pressure ratio (P50/P2)	—
Ps30	Static pressure at HPC	psia
phi	Ratio of fuel flow to Ps30	pps/psi
NRf	Corrected fan speed	rpm
NRc	Corrected core speed	rpm
BPR	Bypass Ratio	—
farB	Burner fuel-air ratio	—
htBleed	Bleed Enthalpy	—
Nf_dmd	Demanded fan speed	rpm
PCNfR_dmd	Demanded corrected fan speed	rpm
W31	High Pressure Turbine coolant bleed	lbm/s
W32	Low Pressure Turbine coolant bleed	lbm/s

**Table 2:** Hyperparameters of all models used to test the new loss function presented in Section 3.1.

Deep Learning Architecture	Hyperparameters
Bi-LSTM	Number of layers: 2
	Layer 1 units: 100 Layer 2 units: 50
	Activation function: Leaky ReLU
DNN	Number of layers: 6
	Layer 1 units: 100 Layer 2 units: 500 Layer 3 units: 100 Layer 4 units: 250 Layer 5 units: 12 Layer 6 units: 6
	Activation function: ReLU
CNN1D	Number of layers: 2
	Layer 1 units: 64 Layer 2 units: 64
	Activation function: ReLU
	Filter size: 3 x Features
Bi-GRU	Number of layers: 2
	Layer 1 units: 100 Layer 2 units: 50
	Activation function: Leaky ReLU

**Table 3:** Number of instances and percentage of minority class in training and testing data of air pressure system (APS) failure dataset.

Data	Number of Positive Instance	Number of Negative Instance	Percentage of Minority Class
Training	1000	59,000	1.67%
Testing	375	16,000	2.34%

**Table 4:** Hyperparameters of all models used to test the focal loss function presented in Section 3.2.

Deep Learning Architecture	Hyperparameters
Bi-LSTM	Number of layers: 2
	Layer 1 units: 32 Layer 2 units: 16
	Activation function: ReLU
DNN	Number of layers: 2
	Layer 1 units: 64 Layer 2 units: 64
	Activation function: Sigmoid
CNN1D	Number of layers: 1
	Layer 1 units: 30
	Activation function: ReLU
	Filter size: 10 × 1
Bi-GRU	Number of layers: 2
	Layer 1 units: 32 Layer 2 units: 16
	Activation function: ReLU

**Table 5:** Scoring function and root mean squared error (RMSE) before and after using dynamic weighting (DW) for loss function while maintaining the architecture of deep learning models. Blue colored text indicates improved performance while red colored text indicates worsened performance. The values in this table are the median values across 20 experimental runs.

Deep Learning Architecture	Scoring Function	RMSE
Bidirectional LSTM	178.568	20.1
Bidirectional LSTM + DW	129.089	13.9
	−27.7%	−30.6%
DNN	93,473.3	23.1
DNN + DW	13,741.3	23.9
	−85.2%	+3.4%
CNN1D	112.858	22.3
CNN1D + DW	63.002	21.1
	−44.1%	−5.7%
Bidirectional GRU	169.550	11.6
Bidirectional GRU + DW	81.899	12.9
	−51.6%	+11.8%

**Table 6:** Results of cost, false negative rate, false omission rate, and recall using Bi-LSTM, DNN, CNN1D, and Bi-GRU with and without FL. Blue colored text indicates improved performance. The values in this table are the median values across 20 experimental runs.

Deep Learning Architectures	Cost	False Negative Rate	False Omission Rate	Recall
Bidirectional LSTM	22,565	0.101	0.00248	0.898
Bidirectional LSTM + FL	15,160	0.045	0.00113	0.954
	−32.8%	−55.4%	−54.4%	+6.2%
DNN	31,505	0.156	0.00378	0.844
DNN + FL	24,200	0.112	0.00273	0.888
	−28.2%	−39.3%	−27.8%	+5.0%
CNN1D	16,855	0.067	0.00164	0.933
CNN1D + FL	12,580	0.012	0.00030	0.988
	−25.4%	−82.1%	−81.7%	+5.9%
Bidirectional GRU	35,480	0.177	0.00429	0.822
Bidirectional GRU + FL	21,350	0.074	0.00187	0.925
	−39.8%	−58.1%	−56.4%	+12.5%
